# Important Announcement

**Published:** 1918-12

**Authors:** A. L. Benedict


### BUFFALO MEDICAL JOURNAL

**A Monthly Review of Medicine and Surgery**

Owing to the uncertainty as to the duration of military service and the comparatively frequent change of stations, the retiring editor, Dr. A. L. Benedict, requests that all communications regarding the Journal under his management, which ceases between the December and the January issue, be directed to his permanent forwarding address, 126 N. Pearl St., Buffalo.

Yearly Volume 74 DECEMBER, 1918 Number 5

### Important Announcement!

Beginning with the next, January 1919, issue, this journal will be taken over by and merged with the **Medical Review of Reviews**. Thus, for a second time, it goes to New York, the first removal having occurred when the first Editor and Founder, Dr. Austin Flint, changed his residence from Buffalo.

In vacating the Editorial Chair, it may be allowable to speak quite frankly and personally. Shortly after the death of Lt. Col. William Warren Potter in March 1911, the suggestion of assuming the management of the Buffalo Medical Journal came from several sources. In spite of considerable experience with various forms of editorial writing, especially with the Medical and Surgical Reporter of Philadelphia, the Medical News and the Medical Times of New York, the idea of assuming the entire responsibility for a journal was somewhat alarming. After assisting in the conduct of the Journal for six months and trying out on a small scale the prosaic but extremely important duties on which the success of any periodical depends, after having tried to induce others to take up the work which Dr. Potter and, during the last part of his life, Miss Margaret Bowden, had carried on from 1888, it was decided either to carry on or wind up the Buffalo Medical Journal.

It may be frankly stated that this decision depended largely on a personal veneration for antiquities and the realization that the handicap of America in this respect can be overcome only by carefully preserving institutions that are half a century or more old. As **only two other** medical journals extant on the American Continent surpassed the Buffalo Medical Journal in age, local pride added to the former consideration.

For a magazine of local scope and whose excuse for existence required it to remain local, this Journal has been a decided success throughout the seventy-four years of its existence. This success has been due to the very cordial cooperation and support of the profession of New York State and of the firms to which any periodical must look for its main financial support. Of course, the ambition was maintained to go very far beyond local usefulness and it was somewhat disappointing that Buffalo was not regarded more as a medical center. However, it is particularly gratifying to learn from the Publishers of the **Medical Review of Reviews,** a publication of national scope and circulation, that this Journal has not only been unusually successful as a local medium but has measured up to the standards of many of general scope.

With nine of the thirteen members of our editorial staff in military service, the continuance of publication for the last twelve months has been possible only because of the unusual ability and willingness of two persons, Mr. Alf. E. Tovey, the printer, and Mrs. A. L. Benedict, to assume burdens naturally devolving upon the managing editor. The gradual increase of demands of the service up to a standard usually considered impossible in civil life, has correspondingly reduced the opportunity to maintain a reserve of material for publication or even to advise as to matters of general nature. Even before the war attempts were made to interest others so as to afford insurance against too great dependence on one life. These attempts having failed in Buffalo, the kind offer from New York to preserve the identity of the Buffalo Medical Journal with the progressive **Medical Review of Reviews,** has been accepted.

It is highly gratifying that the surrender of complete local maintenance of the Journal is the direct result of war conditions and not even in the economic sense of serious impairment of either subscriptions or advertising, such as usually leads to similar mergers.

The Buffalo Medical Journal passes into new hands in a prosperous state with its quality unimpaired, rather enhanced by the fact that it has had to depend for the last year, even more than normally, upon the collaboration of many of its friends.

Most of our readers are familiar with the **Medical Review of Reviews,** which, being a much larger magazine, contains many features not contained in the Buffalo Medical Journal. One of the special features of the **Review** is its Index Medicus, which lists each month the titles of every medical article published in practically every medical journal in the world, together with the author's name, date and place of the publication, etc. The **Review** is the only publication in the world containing such a department. It is also the only publication which contains a department devoted to diseases of old age.

Beginning with the January issue, when the Buffalo Medical Journal will be absorbed by the **Medical Review of Reviews**, the combined journal will contain 112 pages monthly and promises to offer to the medical profession the best to be had in American Medical literature. The subscription price remains $2.00 yearly and we sincerely trust that every subscriber to this Journal will renew with the **Medical Review of Reviews**, 206 Broadway, New York. Unexpired subscriptions will of course also be filled by the combined journal.

We bespeak for the new management the same cordial and generous support that the journal has enjoyed in the past.

A. L. BENEDICT.

